# An Alternative Mode of GPCR Transactivation: Activation of GPCRs by Adhesion GPCRs

**DOI:** 10.3390/ijms26020552

**Published:** 2025-01-10

**Authors:** Hsi-Hsien Lin

**Affiliations:** 1 Department of Microbiology and Immunology, Graduate School of Biomedical Sciences, College of Medicine, Chang Gung University, Taoyuan 33302, Taiwan; hhlin@mail.cgu.edu.tw; Tel.: +886-03-2118800-3321; 2 Center for Molecular and Clinical Immunology, College of Medicine, Chang Gung University, Taoyuan 33302, Taiwan; 3 Department of Anatomic Pathology, Chang Gung Memorial Hospital-Linkou, Taoyuan 33305, Taiwan; 4 Division of Rheumatology, Allergy and Immunology, Chang Gung Memorial Hospital-Keelung, Keelung 20401, Taiwan

**Keywords:** adhesion GPCR, GPCR transactivation, protease, receptor, signaling

## Abstract

G protein-coupled receptors (GPCRs), critical for cellular communication and signaling, represent the largest cell surface protein family and play important roles in numerous pathophysiological processes. Consequently, GPCRs have become a primary focus in drug discovery efforts. Beyond their traditional G protein-dependent signaling pathways, GPCRs are also capable of activating alternative signaling mechanisms, including G protein-independent signaling, biased signaling, and signaling crosstalk. A particularly novel signaling mode employed by these receptors is GPCR transactivation, which enables cross-communication between GPCRs and other receptor types. Intriguingly, GPCR transactivation by distinct GPCRs has also been identified. In this review, I provide an overview of the known GPCR transactivation mechanisms and explore recently uncovered GPCR transactivation mediated by adhesion-class GPCRs (aGPCRs). These aGPCR-GPCR transactivation processes regulate unique cell type-specific functions, offering an exciting opportunity to develop therapies that precisely modulate specific GPCR-mediated biological effects.

## 1. Introduction

G protein-coupled receptors (GPCRs) are cell surface proteins characterized structurally by a common seven-pass transmembrane (7TM) framework [1,2]. More than 800 different genes in the human genome are predicted to encode functional GPCRs. Due to their abundance, GPCRs constitute the largest family of surface receptors in humans and play a pivotal role in cellular communication and signal transduction [1,2]. These receptors respond to a diverse array of extracellular signals, such as photons, ions, amino acids, peptides, hormones, neurotransmitters, and sensory stimuli [1,2,3]. GPCR-mediated intracellular signaling regulates numerous physiological processes, including sensory perception, chemotaxis, immune responses, neurotransmission, and cellular metabolism. Accordingly, many human pathological conditions, including cancer, are directly caused by GPCR mutation and/or dysfunction, making GPCRs one of the major classes of molecules targeted for drug development [1,2,3,4,5].

GPCR activation is typically initiated by a conformational change in the 7TM region, which occurs after specific ligands bind to the extracellular domain (ECD) or to a groove formed by the transmembrane helices [1,2,3]. Ligand-bound GPCR then functions as a guanine-nucleotide exchange factor and couples to the heterotrimeric Gαβγ protein complex to induce downstream signaling by facilitating the exchange of GDP for GTP on the Gα subunit [2,6]. GTP-bound active Gα protein separates swiftly from the Gβγ dimer, and each dissociated G protein is able to interact with different signaling effectors such as adenylyl cyclase, phospholipase C, and ion channels [6].

In addition to the canonical G protein-dependent signaling, GPCRs are also capable of initiating G protein-independent signaling pathways. Indeed, soon after ligand-induced activation, the GPCR is usually modulated by G protein-coupled receptor kinases (GRKs) for phosphorylation at its intracellular loops and/or C-terminal tail, eventually leading to receptor internalization and desensitization via the recruitment and specific binding of β-arrestins [7,8]. Interestingly, β-arrestins not only prevent further G protein-dependent signaling by desensitizing GPCRs but also serve as scaffolds for alternative signaling pathways, including those involving mitogen-activated protein kinases (MAPKs) [7,8]. In addition to β-arrestins, signaling that is independent of both G proteins and phosphorylation, mediated by other effector molecules such as PDZ domain-containing scaffold proteins and 14-3-3, has also been identified [9,10]. As such, GPCR signaling can be propagated in a multidimensional fashion from various subcellular locations [11,12,13]. The identification of the G protein-independent signaling modes has broadened the functional landscape of GPCRs and led to the idea of biased agonism or ligand bias [14,15,16,17]. Biased agonism is defined as the selective ability of different ligands (agonists) acting at the same GPCR to stabilize unique active conformations to trigger G protein-dependent and/or G protein-independent pathways preferentially [15,17]. Therefore, biased agonism can result in divergent signaling outcomes from a given GPCR, offering significant opportunities for drug design and therapeutic intervention.

GPCR signaling became even more intricate with the discovery of an unconventional signal relay process called GPCR transactivation, a term introduced by Axel Ullrich and colleagues in 1996 [18]. This novel activation mechanism aligns well with earlier findings showing that both G proteins and tyrosine kinases are essential for GPCR-mediated activation of MAPK signaling pathways [19,20,21]. Traditionally, GPCR transactivation refers to the indirect activation of a different receptor type, typically the receptor tyrosine kinases (RTKs), by specific GPCR ligands [22,23]. Interestingly, later studies have shown that some GPCRs can also be transactivated by different types of receptors such as RTKs [24]. These unique transactivation mechanisms allow GPCRs to crosstalk to other receptor families bidirectionally to work in concert to regulate and diversify the signaling outputs and biological responses. The topic of GPCR transactivation has been extensively reviewed recently [24,25,26,27,28], and a detailed discussion of it falls beyond the scope of this article. In this review, I summarize the various mechanisms and functional implications of GPCR transactivation, with a particular focus on the recent identification of novel GPCR transactivation processes mediated by a distinct GPCR subtype, the adhesion-class GPCRs (aGPCRs) [29]. The aGPCR-mediated GPCR transactivation processes discussed here offer novel insights into an unconventional mode of GPCR activation, potentially broadening the scope for developing innovative therapeutic strategies.

## 2. GPCR Transactivation of RTKs and Other Receptor Types

GPCRs and RTKs are two principal families of cell surface receptors critical for cellular communication and signaling [25,26]. Unlike GPCRs, RTKs normally contain an N-terminal ECD for ligand binding, a single-pass transmembrane domain, and an intracellular tyrosine kinase domain [30,31]. The activation of RTKs starts with the binding of soluble protein ligands, primarily growth factors like epidermal growth factor (EGF) and vascular endothelial growth factor (VEGF). This ligand binding induces receptor dimerization, initiating auto-transphosphorylation of tyrosine residues within the intracellular C-terminal region. Receptor tyrosine phosphorylation then serves as a cue to recruit and activate various signaling adaptor proteins, including Src kinase (Src), phosphoinositide 3-kinases, and phospholipase C, thereby initiating downstream signaling cascades [30,31].

**Figure 1:**
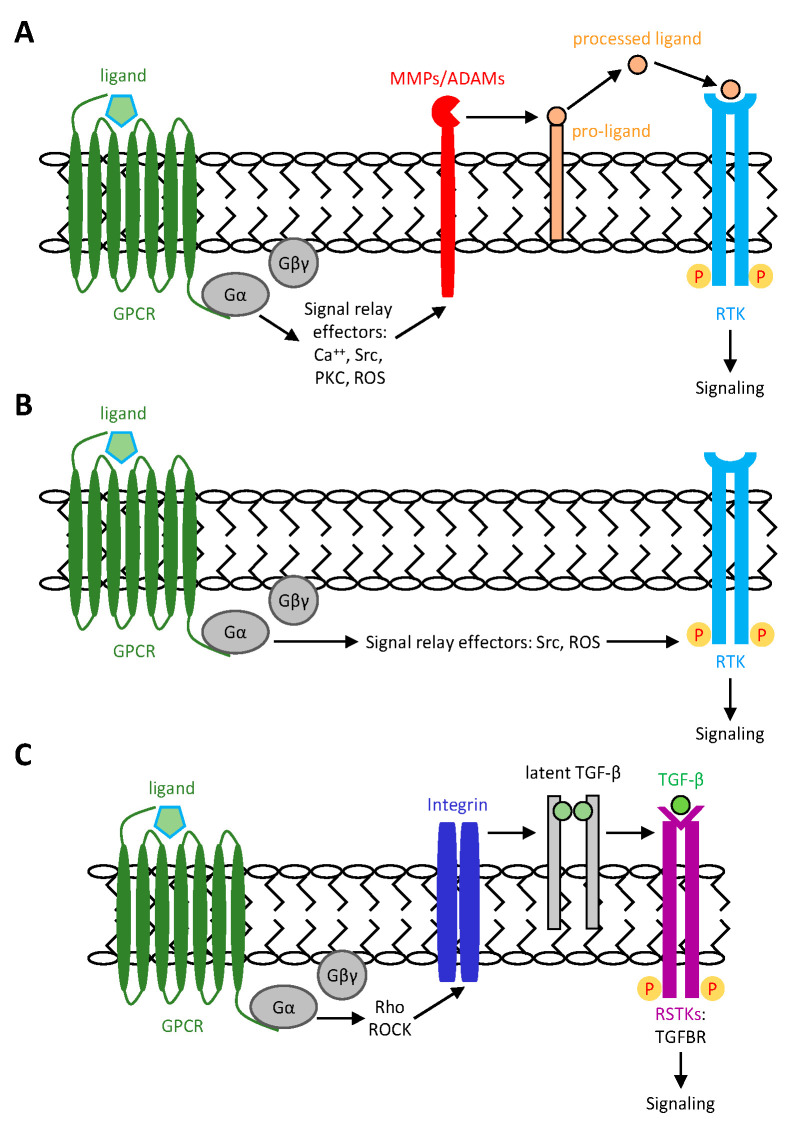
Schematic representations of GPCR transactivation of cell surface receptors. (**A**) Ligand-dependent triple-membrane-passing-signal (TMPS) mechanism and (**B**) ligand-independent mechanism of GPCR transactivation of RTKs. Binding of a GPCR-specific ligand induces heterotrimeric G protein activation, leading to activated signaling effector proteins. In (**A**), these GPCR-induced signaling effectors elicit the activation of MMPs or ADAMs, cleaving RTK ligand pro-forms that are located on the cell membrane or extracellular matrix. Released active RTK ligand(s) then binds to specific RTKs to trigger receptor auto-transphosphorylation and subsequent downstream signaling. In (**B**), GPCR-activated effector proteins induce RTK auto-transphosphorylation and signaling directly via intracellular crosstalk without activating MMPs or ADAMs; hence, there is no need for RTK ligand(s). (**C**) Ligand-dependent mechanism of GPCR transactivation of RSTKs, such as TGFBR1. In this case, GPCR activation induces a RhoA/ROCK-dependent pathway to trigger an integrin-mediated activation of the latent TGF-β complex on the cell surface. Active TGF-β eventually binds to TGFBR1 to induce receptor activation and signaling.

GPCRs and RTKs have traditionally been regarded as two distinct signaling entities, with GPCRs primarily involved in regulating cell metabolism, while RTKs are mainly responsible for controlling cell growth and differentiation [1,2,30,31]. However, an unexpected result by Ullrich et al. showed a novel cross-communication of the two receptor systems, i.e., GPCR transactivation of RTK. Briefly, a rapid and transient tyrosine phosphorylation of the epidermal growth factor receptor (EGFR) was detected readily in Rat-1 fibroblast cells when stimulated by a number of GPCR-specific agonists [18]. Since this observation, many different RTKs including EGFR, VEGFR and platelet-derived growth factor receptor (PDGFR), and other receptor types such as the receptor serine/threonine kinases (RSTKs) and Toll-like receptors (TLRs) have been found to be transactivated by numerous GPCRs via diverse mechanisms (Figure 1) [22,23,24,25,26,27,28,32]. The different molecular mechanisms and functional implications of GPCR transactivation are summarized below.

### 2.1. Ligand-Dependent Transactivation Mechanism

This mechanism is also named ligand-dependent triple-membrane-passing-signal (TMPS) mechanism and involves the cleavage and shedding of membrane-bound growth factor precursors by active metalloproteases (Figure 1A) [24,25,26]. Indeed, the TMPS mechanism typically involves the activation of surface matrix metalloproteinases (MMPs) or a disintegrin and metalloproteinases (ADAMs) by GPCR-activated signaling effectors, such as Src. Activated MMPs and ADAMs then cleave growth factor precursors, such as heparin-binding EGF-like growth factor (HB-EGF), neuregulin, and amphiregulin that are localized on the cell surface or extracellular matrix (ECM). Finally, processed active growth factor ligands are shed and bind to RTKs to induce the downstream signaling pathways. In this model, signals from a GPCR agonist traverse the cell membrane three times, involving different effector molecules, to induce RTK activation [24,25,26]. Many GPCR-activated effectors, including intracellular Ca^2+^, protein kinase C (PKC), Src, and reactive oxygen species (ROS), are all implicated in the signal relay processes for the activation of MMPs/ADAMs and RTKs [24,25,26].

### 2.2. Ligand-Independent Transactivation Mechanism

In certain cases, no shedding of growth factor ligands was observed in GPCR-mediated RTK transactivation, suggesting the involvement of ligand-independent processes in RTK activation [24,25,26]. In this mechanism, intracellular signaling crosstalk between GPCRs and RTKs, mediated by distinct relay effectors, leads to the phosphorylation of the cytoplasmic domain of RTKs, triggering receptor activation (Figure 1B). Several signaling effectors including ROS and intracellular tyrosine kinases such as Src family proteins have been shown to play a role in the ligand-independent transactivation of RTKs by GPCRs [24,25,26,27]. In some cases, GPCRs can directly interact with RTKs to form a dimeric complex, thereby modulating RTK activity either positively or negatively [24,25,26,27].

### 2.3. GPCR Transactivation of the Receptor Serine/Threonine Kinases (RSTKs) and Toll-like Receptors (TLRs)

In addition to RTKs, GPCR transactivation of surface receptors has been extended to include members of the receptor serine/threonine kinase receptor (RSTK) family, especially the transforming growth factor-β receptor (TGFBR) molecules (Figure 1C) [26,33]. Stimulation of vascular smooth muscle cells (VSMCs) with thrombin or endothelin-1 leads to a transient increase in Smad2 phosphorylation, which is the major downstream effector of TGFBR1 [34,35,36,37]. Interestingly, GPCR transactivation of TGFBR1 in VSMCs was shown to involve the RhoA/ROCK-dependent integrin-mediated activation of the latent TGF-β complex on the cell surface (Figure 1C) [26,34,38,39], strikingly similar to the ligand-dependent TMPS mechanism of GPCR transactivation of RTKs.

Emerging evidence also indicates that GPCR activation can trigger the transactivation of TLRs, the prototypic pattern recognition receptors (PRRs) essential for mediating innate immune responses [24,40,41]. The desialylation activity of sialidase 1, also known as neuraminidase 1 (Neu1), was found to be critical for the dimerization and activation of TLR4 induced by its microbial ligand, LPS [42]. Interestingly, Neu-1 formed a complex with MMP9 to interact with TLR4 on the surface of macrophages [43]. Abdulkhalek et al. later showed that treatment of macrophages with various GPCR agonists such as bombesin, bradykinin, and lysophosphatidic acid (LPA) upregulates Neu1 activity and induces NFκB activation, which was the downstream effector of TLR4. Importantly, the enhanced Neu1 activity induced by these GPCR agonists was dependent on the activity of G proteins and MMP9. Moreover, the bombesin receptor, neuromedin B receptor (NMBR), associated with TLR4 and MMP9 to form a GPCR signaling platform to promote the Neu1-MMP9 crosstalk that is critical for TLR activation and signaling [40,43]. Taken together, GPCR transactivation of various receptor types, including RTKs, RSTKs, and TLRs, has been identified to be mediated through a variety of distinct mechanisms.

### 2.4. GPCR Transactivation by RTKs

**Figure 2:**
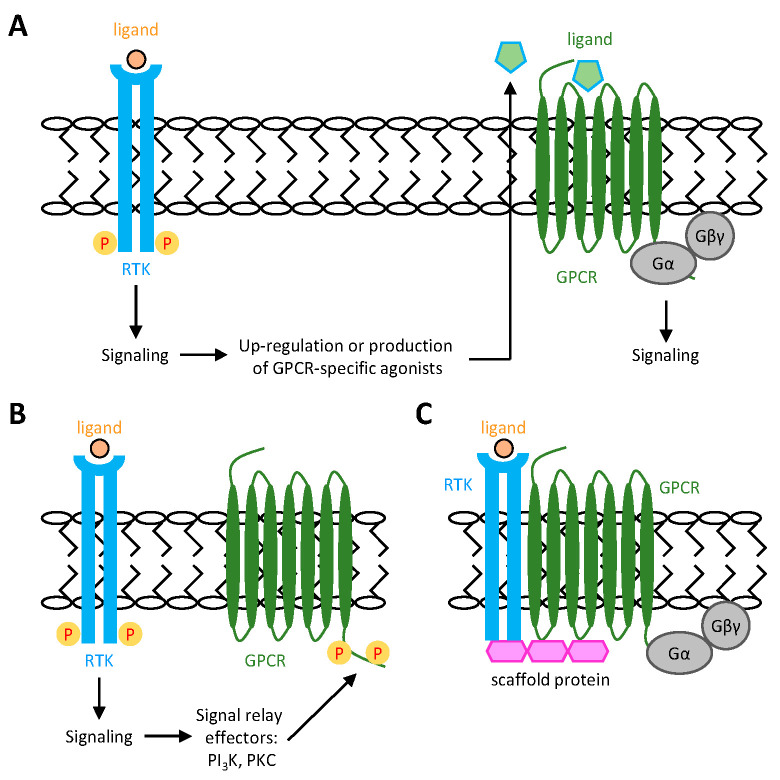
Schematic representations of GPCR transactivation by RTKs. (**A**) Ligand-dependent mechanism and (**B**,**C**) ligand-independent mechanism of GPCR transactivation by RTKs. (**A**) Activation of RTKs by unique agonists can induce the production and release of specific GPCR ligands, which subsequently bind and activate GPCRs. On the other hand, RTK activation can activate GPCRs directly by inducing phosphorylation of cytoplasmic residues of GPCRs via intracellular crosstalk with RTK-activated signaling effectors (**B**) or by the formation of GPCR-RTK complex mediated by intracellular scaffold proteins (**C**).

Given the highly versatile crosstalk between GPCRs and RTKs, it is not surprising that RTKs can also reciprocally transactivate GPCRs [24,44]. Similar to the GPCR transactivation of RTKs, both ligand-dependent and ligand-independent mechanisms of RTK-mediated GPCR transactivation were identified (Figure 2). In the ligand-dependent mechanism, GPCR transactivation is usually achieved through the production and secretion of the GPCR-specific ligand via the RTK-mediated signaling (Figure 2A). For example, the activation of many RTKs, including EGFR, PDGFR, VEGFR, and nerve growth factor receptor (NGFR), can induce the biosynthesis and secretion of sphingosine 1-phosphate (S1P), which then acts in an autocrine or paracrine manner to activate sphingosine-1-phosphate receptor 1 (S1PR1) [45,46,47]. In the ligand-independent mechanism, GPCR transactivation is induced through GPCR phosphorylation driven by various RTK-activated signaling effectors, as well as through the formation of GPCR-RTK complexes via direct protein-protein interactions or intracellular scaffolding proteins (Figure 2B,C) [45,48]. Interestingly, recent evidence suggests that upon growth factor stimulation, active RTKs can directly interact with Gαi to promote its phosphorylation and activation, potentially bypassing the involvement of GPCRs [49].

## 3. GPCR Transactivation by GPCRs

The findings presented above clearly demonstrate that GPCR-mediated responses can drive the transactivation of various key cell surface receptors. Hence, it is entirely plausible that the MMP-mediated transactivation of RTKs by GPCRs has evolved to enable the activation of distinct GPCR subtypes. Essentially, the transactivation of one GPCR by another

**Figure 3:**
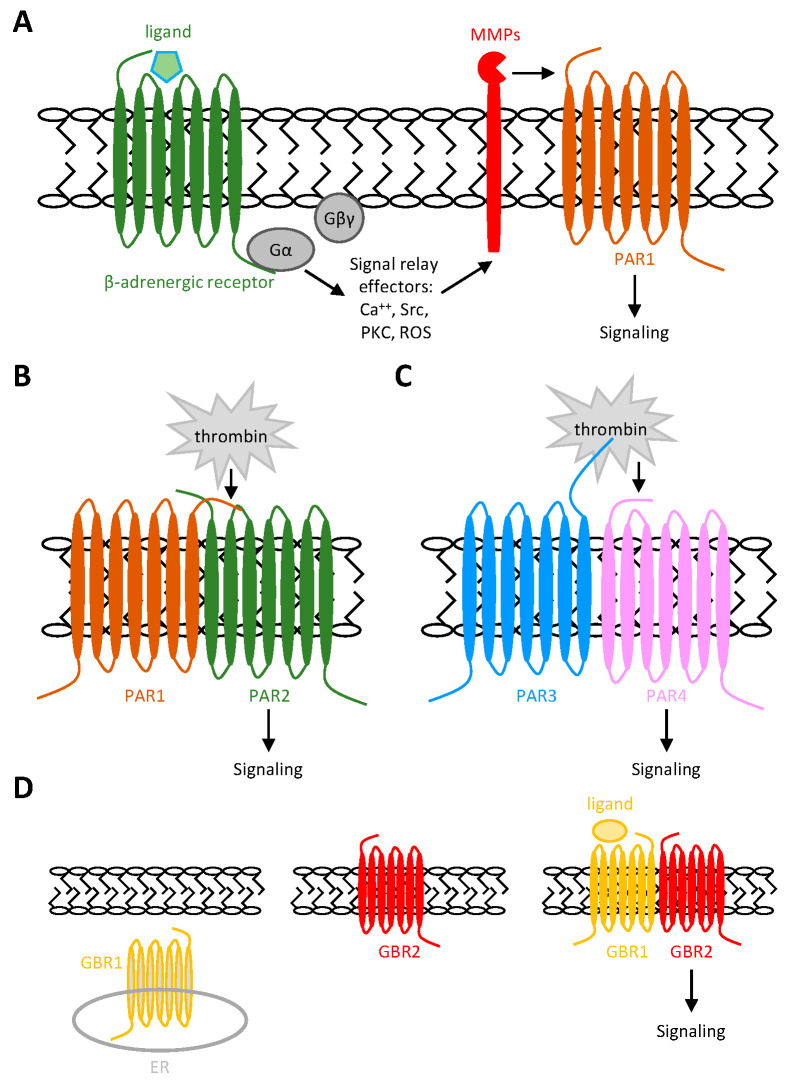
Schematic representations of GPCR transactivation by GPCRs. (**A**) MMP-dependent transactivation mechanism of PAR1 by βAR. As a result of βAR activation and signaling, MMP13 is activated to induce PAR1 activation by targeting and cleaving PAR1. (**B**–**D**) GPCR transactivation by heterodimeric GPCR-GPCR complexes. In (**B**), PAR1 mediates PAR2 transactivation via the formation of PAR1-PAR2 heterodimer. In this case, PAR2 is activated by the tethered agonistic peptide of PAR1 generated by thrombin digestion. In (**C**), mPAR4 is activated by thrombin that is associated with the neighboring mPAR3 cofactor. In (**D**), GBR2 heterodimerizes with GBR1 to transport the normally intracellular GBR1 to the cell membrane to act as the dominant GABA receptor. In contrast, GBR2 functions as the main signaling receptor to induce G protein activation following the binding of GABA to GBR1.

Of particular interest, many instances of GPCR-GPCR transactivation are focused on, but not limited to, protease-activated receptors (PARs), which are discussed here as the primary examples. PARs are special GPCRs that are activated by a tethered agonistic peptide unmasked after the proteolysis of their extracellular N terminus by specific proteases [50,51,52]. Four PAR family members, namely, PAR1, 2, 3, and 4, can be identified in humans, and the most well-known protease activators of PARs are thrombin, which cleaves PAR1, PAR3, and PAR4, and trypsin, which activates PAR2 [50,51,52,53]. Nevertheless, apart from the canonical PAR-mediated signaling pathways induced by these renowned proteases, PAR-mediated biased signaling was triggered upon their cleavage by other proteolytic enzymes like activated protein C, tryptase, and MMPs [51,52]. For example, MMP1, MMP2, and MMP13 have been shown to digest PAR1 at alternative positions distinct from the thrombin-cutting site to induce biased signaling pathways [54,55,56]. Interestingly, Blaxall et al. demonstrated that the stimulation of β-adrenergic receptor (βAR) in cardiac fibroblasts and cardiomyocytes led to an upregulation of MMP13, which then induced PAR1 transactivation by cleaving it at an uncanonical site (Figure 3A) [28,57]. Thus, these results identified an MMP13-mediated βAR-induced PAR1 transactivation mechanism that may be involved in the isoproterenol-dependent cardiac dysfunction [57].

Recently, additional mechanisms of GPCR-PAR transactivation beyond MMPs have been identified. O’Brien et al. showed that PAR1 can interact specifically with PAR2 to form a heterodimeric complex on endothelial cells where PAR1 is cleaved normally by thrombin. Unexpectedly, the thrombin-exposed tethered peptide ligand of PAR1 subsequently binds to and activates the neighboring PAR2, which is not a typical substrate of thrombin (Figure 3B) [51,58]. As a result, the thrombin-induced signaling activated Rac1 via the transactivated PAR2 but not RhoA, which is the normal downstream effector of PAR1, in endothelial cells [59]. In this case, the formation and co-trafficking of the PAR1-PAR2 heterodimer is critical for the subsequent PAR2 transactivation by thrombin [60,61]. Homo- and hetero-dimerization of GPCRs such as PARs are very common and play important roles in their biological functions [62]. Beyond the direct transactivation of PAR2 by the tethered peptide ligand of PAR1, it has been demonstrated that one partner in a PAR heterodimer can modulate the signaling of the other by serving as an allosteric modulator or cofactor [62]. For example, murine PAR3 forms a heterodimeric complex with PAR4 on the surface of mouse platelets and works as a cofactor to facilitate murine PAR4 cleavage and activation by thrombin (Figure 3C) [62]. Due to the presence of a “hirudin-like” domain in its N-terminus for promoting thrombin binding, murine PAR3 is a high-affinity thrombin receptor [63]. By contrast, murine PAR4 lacks the “hirudin-like” domain and is a low-affinity thrombin receptor [64]. Interestingly, however, the cleavage of mPAR3 by thrombin alone does not result in receptor activation or signaling [65]. It appears that, although thrombin cleavage does not directly activate mPAR3, the cleaved mPAR3 retains thrombin long enough to facilitate its accessibility to the adjacent mPAR4, enabling its cleavage and activation [66,67]. In humans, a similar phenomenon was noted in the PAR1-PAR4 heterodimer, in which PAR1 acts as a cofactor of PAR4 to promote its rapid cleavage by thrombin [68].

Another famous example of GPCR heterodimer-mediated transactivation was that of the metabotropic γ-amino-butyric acid (GABA)_B_ receptor-1 (GBR-1) and -2 (GBR-2) [69,70]. When expressed individually, GBR-1 is retained intracellularly, while GBR-2 traffics efficiently to the cell surface but fails to bind to GABA. When co-expressed, GBR-1 forms an obligatory heterodimer with GBR-2, a complex essential not only for the surface expression but also for the neuroregulatory function of the heterodimer [71]. Indeed, studies have shown that, upon the co-trafficking and expression of the GBR1-GBR2 heterodimer on the membrane, GBR-1 primarily acts as the main GABA receptor for ligand binding, while GBR-2 is responsible for coupling with G proteins to mediate intracellular downstream signaling (Figure 3D) [71]. Collectively, GPCRs like PARs and GBRs can be transactivated by other GPCRs through a variety of distinct mechanisms, including the MMP-mediated cleavage, direct activation by a tethered peptide of a neighboring GPCR, or cofactoring GPCRs.

## 4. GPCR Transactivation by Adhesion GPCRs

**Figure 4:**
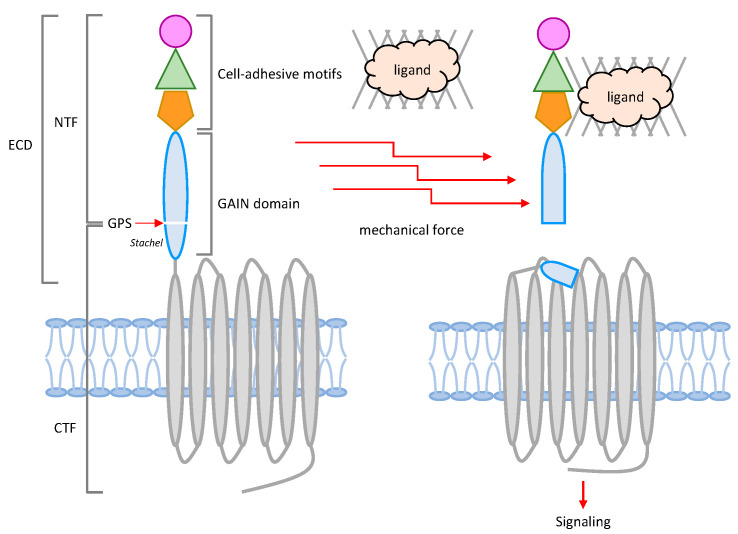
Schematic representation and molecular characteristics of aGPCRs. aGPCRs are characterized by a long extracellular domain (ECD) preceding the 7TM domain. The ECD is structurally divided into two segments: an N-terminal portion containing cell-adhesive protein motifs and a GAIN domain, which includes a conserved GPS (GPCR proteolysis site) motif. GPS auto-proteolysis typically cleaves the receptor molecule into two fragments: an N-terminal fragment (NTF) and a C-terminal fragment (CTF). These fragments normally remain non-covalently associated, forming a dimeric receptor complex on the cell surface. Activation of most aGPCRs is commonly triggered by a tethered agonistic peptide, known as the *Stachel* peptide, which becomes exposed after the dissociation or displacement of the NTF-CTF complex induced through the binding of specific cellular or ECM ligands without or with additional mechanical stimulation.

More recently, atypical GPCR transactivation processes mediated by aGPCR proteins have been identified, further highlighting the growing diversity of GPCR transactivation mechanisms. A total of 33 different aGPCRs, representing the second largest GPCR subfamily, are expressed in humans [29,72]. Importantly, several aGPCRs are causally linked to rare human disorders, such as bilateral frontoparietal polymicrogyria and vibratory urticaria, underlining their biological significance [73,74]. These receptors are unique among the GPCR superfamily, as they are hallmarked by having a much-elongated modular ECD (Figure 4). Notably, the N-terminal region of aGPCR-ECD usually consists of assorted cell-adhesive protein motifs, including the EGF-like domain, lectin-like domain, and immunoglobulin-like (Ig) domain, etc. These protein motifs are followed by a signature GPCR autoproteolysis-inducing (GAIN) domain, which is immediately juxtaposed to the 7TM region. During the biosynthesis of most aGPCRs, an auto-proteolytic reaction occurs at a consensus GPCR proteolysis site (GPS) within the GAIN domain to cleave the receptor into a cell-adhesive N-terminal fragment (NTF) and a 7TM-containing C-terminal fragment (CTF). Interestingly, the receptor is typically expressed as a non-covalent NTF-CTF dual-subunit complex on the cell surface [29,72].

The diverse adhesive protein motifs of the NTF are often responsible for interactions with specific cellular ligands or binding partners, including lipids, polysaccharides, and proteins—particularly cell surface and ECM proteins. Upon the binding of specific ligands, aGPCRs induce G protein-dependent and/or -independent signaling similar to most GPCRs [29,75,76]. Unlike other GPCRs, however, the conventional activation mechanism of many aGPCRs involves some additional steps following ligand binding, a process known as the tethered agonism model. Specifically, ligand binding, either alone or with the assistance of mechanical forces, results in the dissociation or displacement of NTF from the CTF. This allows the GPS-cleaved N-terminal sequence, alternatively named the *Stachel* peptide, of the CTF to be dislodged and inserted into a binding-pocket within the 7TM helices (Figure 4) [77,78,79]. The tethered agonism model of aGPCR activation closely resembles that of PARs but adds an extra layer of regulation through the dissociation or displacement of the NTF and CTF, which is triggered by ligand binding and/or mechanical disturbance. As such, increasing evidence suggests that some aGPCRs may function as mechanosensitive receptors [80,81]. Aside from the tethered agonism activation mechanism, other forms of aGPCR activation, including GPS cleavage-independent and NTF-CTF dissociation-independent modes, have been uncovered. Intriguingly, several aGPCRs have been shown to induce the transactivation of another GPCR subtype, further deepening our understanding of aGPCR functions.

### 4.1. Transactivation of the Frizzled (Fz) Receptor by GPR124/ADGRA2

GPR124/ADGRA2 is a member of the ADGRA group of aGPCRs and was identified originally due to its significant expression in the colorectal cancer vasculature; hence, it was alternatively named the tumor endothelial marker 5 (TEM5) [82]. Four tandem copies of leucine-rich repeat (LRR), a LRR C-terminal domain, an Ig domain, a hormone receptor motif (HRM), plus the GAIN domain, make up its ECD [29]. On the other hand, its intracellular C-terminal region contains a PDZ domain-binding motif, which was shown to bind to the PDZ domain-containing human homolog of the Drosophila discs large tumor suppressor (hDlg) protein [83]. Significant levels of GPR124 were expressed in the endothelium of the central nervous system (CNS) during embryogenesis. Of interest, GPR124-ECD was shed from endothelial cells during angiogenesis and bound to glycosaminoglycans (GAGs) and αvβ3 integrin to promote endothelial cell survival [84]. Collectively, these prior findings suggest that GPR124 plays a regulatory role in both angiogenesis and vasculogenesis [82,85].

Multiple studies utilizing various *Gpr124*-deficient mouse models have firmly established the critical role of GPR124 in CNS angiogenesis, vascular development, and the formation of the blood–brain barrier (BBB) [86,87,88]. In addition, Anderson et al. reported additional developmental abnormalities in the palate and lung of *Gpr124*-null mice [86]. Intriguingly, the CNS phenotypes of *Gpr124*-deficient mice shared striking similarities with those of the *Wnt7a*/*Wnt7b* double-knockout animals. Moreover, the canonical Wnt/β-catenin signaling pathways were well known for their roles in embryonic development and adult tissue homeostasis and have been shown to be critically involved in the normal development of palate and lung, too [89,90,91,92]. These findings implied strongly a functional link between GPR124 and the Wnt morphogens.

The canonical Wnt/β-catenin signaling pathway is mediated by the specific binding of soluble Wnt proteins to the Frizzled (Fz)-class GPCRs and low-density lipoprotein receptor-related protein (LRP)5/6 co-receptors [92]. However, many similar but distinct Wnt ligands are produced, and likewise, numerous different Fz receptors are expressed. Given the highly promiscuous pairing of Wnt ligands and Fz receptors, it has been puzzling how specific cells are able to selectively recognize and respond to a particular Wnt ligand. Interestingly, independent experiments performed by several research teams using genetically manipulated mouse and zebrafish models as well as cell-based signaling assays have shown recently that GPR124 acts as a Wnt7a/b-specific co-stimulator of a Fz/LRP receptor complex [93,94,95]. Unexpectedly, it was later demonstrated that GPR124 facilitated the extracellular interaction between Fz and Wnt7a/b, which is initially bound to the reversion-inducing cysteine-rich protein with Kazal motifs (RECK) [96,97,98,99]. RECK is a glycosylphosphatidylinositol (GPI)-anchored protein with five cysteine-knot (CK) motifs, two epidermal growth factor (EGF)-like domains, and three Kazal motifs in its ECD. RECK was identified initially as a negative regulator of multiple MMPs and implicated a tumor-suppressive role [100,101]. Noticeably, *Reck*-deficient mice were embryonic lethal and showed similar phenotypes as those of *Gpr124*-deficient animals, suggesting a role for RECK in GPR124-mediated functions [91].

**Figure 5:**
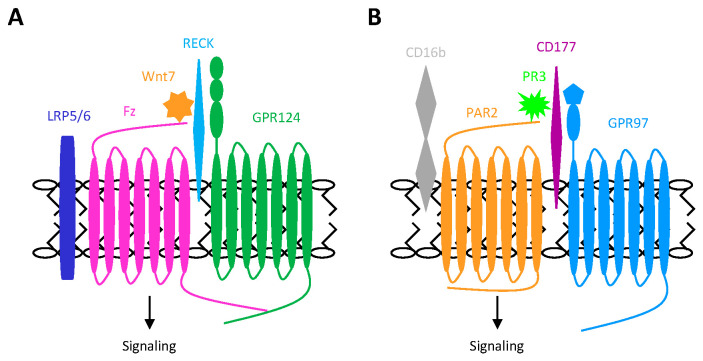
Mechanisms of GPCR transactivation by adhesion GPCRs. (**A**) The transactivation of Fz by GPR124 via the Wnt7/RECK/GPR124/Fz/LRP complex. Briefly, Wnt7a/b binds to the GPI-anchored RECK, and then GPR124 potentiates the RECK-bound Wnt7 to be more accessible to Fz and LRP5/6 co-receptor to induce downstream signaling. (**B**) The transactivation of PAR2 by GPR97 via the PR3/CD177/GPR97/PAR2/CD16b complex. Discharged PR3 binds to the GPI-anchored CD177 to form the mPR3 complex on the neutrophil surface. GPR97 interacts specifically with mPR3 to promote its enzymatic activity, which then cleaves and activates PAR2 to induce inflammatory reactions.

Detailed mechanistic studies have disclosed that the intrinsically unstable Wnt7a/b binds specifically to the CK motifs of RECK via its internal disordered linker region. Interestingly, in the absence of GPR124, the stabilized RECK-bound Wnt7a/b is somehow estranged from the Fz/LRP receptors. In the presence of GPR124, its LRR- and Ig-domains bind to the most N-terminal CK motif of RECK, enhancing the accessibility of RECK-associated Wnt7a/b to Fz/LRP receptors (Figure 5A). In fact, besides interacting with RECK directly, GPR124 also couples to Fz via diverse species-specific mechanisms, highlighting the importance of GPR124 in Wnt7/Fz-mediated signaling. Notably, America et al. showed in zebrafish that Gpr124 and Fz primarily interact through the coupling of intracellular scaffold proteins, such as Dishevelled (Dvl) and Dlg4/Magi3. This interaction involves an internal Dvl-binding motif and a C-terminal ETTV motif located in the intracellular domains (ICDs) of the two GPCRs [98,99,102]. By contrast, mammalian GPR124 exerts both an ETTV-based mechanism and an ICD-independent mechanism mediated by the TM and ECD to interact with Fz, without the help of Dvl [102,103]. Overall, the role of the GPR124/RECK complex in Wnt-specific, Fz-mediated signaling and response is highly conserved across vertebrate evolution, albeit through diverse activation mechanisms.

Therefore, RECK is the dominant specific receptor of Wnt7a/b on endothelial cells and functions as a restraining depot for Wnt7a/b to keep them from interacting with Fz. GPR124, on the other hand, prompts the RECK-associated Wnt7a/b a better exposure to Fz, leading to receptor activation and signaling. In summary, GPR124 facilitates Fz transactivation by promoting the efficient delivery and presentation of surface-bound cryptic Wnt7a/b through a multimeric receptor complex comprising GPR124, RECK, Fz, and LRP5/6. The assembly of GPR124 and GPI-anchored RECK with the Fz/LRP complex on the CNS endothelial cells represents a cell type-specific aGPCR (GPR124)-GPCR (Fz) transactivation mechanism.

### 4.2. Transactivation of PAR2 by GPR97/ADGRG3

GPR97/ADGRG3 belongs to the ADGRG group of aGPCRs and is encoded by the *ADGRG3* gene on a locus at human chromosome 16q21 that also includes genes coding for two other ADGRG members, namely, GPR56/ADGRG1 and GPR114/ADGRG5 [104]. Gpr97 was identified initially as an early lymphocyte-specific gene, *Pb99*, in rodents, where its expression was detected in pre-B lymphocytes and thymocytes but not mature B and T cells [105]. Further studies using *Gpr97*-deficient mice have indicated that Gpr97 may be involved in regulating the fate decision of murine B lymphocytes within secondary lymphoid tissues [106]. Additionally, it appears to play a role in the pathogenic mechanisms underlying acute kidney injury, experimental autoimmune encephalomyelitis, renal interstitial fibrosis, and obesity-associated macrophage inflammation [74].

Contrary to murine Gpr97, we showed previously that human GPR97 is expressed mostly by neutrophils and eosinophils but not lymphocytes [104,107]. Moreover, upregulated GPR97 expression was found in the blood and tissue-infiltrating neutrophils of patients with systemic inflammatory disorders. The involvement of GPR97 in modulating the antimicrobial activity of human neutrophils was demonstrated through the use of agonistic anti-GPR97 mAbs in several in vitro functional assays [107]. These results suggested a clear link between GPR97 and neutrophil-mediated innate immune responses. However, the ligand or binding partner of GPR97 remained unidentified, leaving the mechanism through which GPR97 modulated neutrophil functions largely unknown.

More recently, in an attempt to deorphanize GPR97, we identified a unique GPR97-mediated PAR2 transactivation mechanism in human neutrophils (Figure 5B) [108]. We first detected the exclusive presence of a putative surface ligand for GPR97 on neutrophils, which were rapidly activated upon binding of soluble GPR97-NTF to this ligand. However, the activated neutrophil phenotypes were abolished in the presence of various serine protease inhibitors. Ultimately, we identified membrane-associated proteinase 3 (mPR3) as the interacting protein ligand of GPR97. PR3 is the dominant serine protease of azurophilic granules, and mPR3 is formed by the specific binding of exocytosed PR3 to the GPI-anchored CD177 receptor, a neutrophil-restricted cell surface protein. Notably, the formation of mPR3 somehow results in a significant reduction in its enzymatic activity. We demonstrated that the binding of GPR97 to mPR3 enhances its enzymatic activity, which in turn cleaves and activates PAR2 specifically, triggering rapid neutrophil activation. Concisely, these results indicate that PAR2 is transactivated by GPR97 via the augmented mPR3 activity. Most intriguingly, GPR97-mediated PAR2 transactivation was strictly dependent on the formation of a multi-receptor complex on the neutrophil surface, consisting of GPR97, CD177, PAR2, and CD16b. This complex was surprisingly similar to the GPR124/RECK/Fz/LRP5/6 complex [108].

Accordingly, we propose that, upon formation of the PR3/CD177/GPR97/PAR2/CD16b complex, GPR97 specifically interacts with mPR3, acting as an allosteric modulator to enhance its proteolytic activity. The activated mPR3 subsequently cleaves nearby PAR2, initiating intracellular signaling and driving the inflammatory activation of neutrophils (Figure 5B). The role of CD16b in the GPR97-PAR2 transactivation process is not yet fully understood, but it likely serves as an accessory protein or co-receptor. Critically, PR3 is the autoantigen in autoimmune granulomatosis with polyangiitis (GPA), a condition characterized by the inflammatory necrosis of small-to-medium-sized blood vessels, which results from inappropriate neutrophil activation triggered by the binding of PR3-specific antineutrophil cytoplasmic antibodies (ANCAs) [109]. Interestingly, upregulated GPR97 expression was observed in neutrophils from patients with various inflammatory disorders, including those in the active GPA disease group. Additionally, the expression levels of GPR97 were positively correlated with ANCA titers in GPA patients [108]. Therefore, GPR97 is likely involved in the pathogenesis of GPA by binding to and modulating mPR3 on neutrophils. Notably, PAR2 is the primary PAR protein expressed on human neutrophils, while the GPI-anchored CD177 and CD16b are almost exclusively found on these cells. In summary, the PR3/CD177/GPR97/PAR2/CD16b complex constitutes a highly unique inflammatory effector apparatus, driving the human neutrophil-specific aGPCR (GPR97)-GPCR (PAR2) transactivation process. Intriguingly, the membrane-associated serine protease mPR3 plays a critical role in this transactivation mechanism, akin to the function of MMPs or ADAMs in the canonical ligand-dependent TMPS mechanism of GPCR transactivation of RTKs.

### 4.3. Functional and Therapeutic Implications of GPCR Transactivation

GPCR transactivation serves several critical functions in cellular signaling [16,24,26,27]. First, it enables the amplification of signals from various receptor types, resulting in robust and sustained cellular responses—an essential feature in processes like cell proliferation, migration, and differentiation. Second, it provides a mechanism for fine-tuning cellular responses by modulating the intensity and duration of intracellular signaling. Lastly, GPCR transactivation facilitates the integration of diverse extracellular signals into a unified cellular response. This ability to consolidate signals is vital for complex physiological processes such as immune responses, tissue repair, and neural communication.

Given the critical roles of GPCRs and RTKs in numerous pathophysiological processes and their prominence as major therapeutic targets, a deeper understanding of GPCR transactivation holds significant promise for clinical applications, especially in the design of innovative therapeutic strategies. Modulating GPCR transactivation of RTKs could help regulate intricate signaling networks implicated in widespread diseases such as cancer, cardiovascular conditions, and neurodegenerative disorders. For instance, selective inhibitors targeting EGFR transactivation by GPCRs could potentially suppress tumor growth and metastasis, complementing existing therapies that directly target RTKs.

The inclusion of multiple receptor components in aGPCR-mediated GPCR transactivation significantly broadens the spectrum of potential target molecules, expanding the opportunities for functional modulation and therapeutic implication. For instance, the Gpr124/Reck receptor complex has been effectively targeted with a BBB-specific recombinant Wnt agonist to address BBB dysfunctions commonly associated with various neurological disorders [110,111]. Additionally, GPR124-mediated Wnt7-β-catenin activation has been shown to drive the formation of brain metastases originating from CD44^+^ cancer stem cell-derived pericyte-like cells through enhanced trans-endothelial migration. Notably, disrupting this GPR124-mediated activation mechanism—either by silencing GPR124 and Wnt7b or using a potent Wnt/β-catenin-specific small-molecule signaling inhibitor—led to a significant reduction in brain metastases of lung adenocarcinoma cells [112]. In summary, GPCR transactivation represents a complex and versatile mechanism through which GPCRs influence diverse signaling networks. By activating RTKs and other receptor types, GPCRs can enhance, regulate, and coordinate cellular responses, underscoring their pivotal roles in both normal physiology and disease progression. A deeper understanding of the activation mechanisms and signaling cascades involved in GPCR transactivation will pave the way for the development of future therapeutics.

## 5. Conclusions and Perspectives

GPCR transactivation is one of the versatile signaling mechanisms used by GPCRs to amplify, fine-tune, integrate, and modulate diverse intracellular signaling networks for important functional responses. The two examples of aGPCR-mediated GPCR transactivation discussed here illustrate a unique and unconventional GPCR-GPCR transactivation mechanism, distinct in several ways. Firstly, these transactivation mechanisms are specifically mediated by distinct aGPCR molecules, leading to the selective activation of a unique member of a different GPCR subfamily. Secondly, multiple cell surface proteins are needed to form the cell type-specific functional aGPCR-GPCR receptor complex for the transactivation reaction. Thirdly, the long N-terminal ECD of aGPCR appears to primarily function as an activating cofactor (or allosteric activator) of a receptor-bound soluble effector molecule, which in turn targets an adjacent GPCR partner.

In addition to representing a novel GPCR transactivation mechanism, these distinctive processes have uncovered an unexpected functional role for aGPCRs as “trans-activators” of distinct GPCRs. This function diverges significantly from the prevailing consensus that most aGPCRs operate through *Stachel* peptide-mediated 7TM signaling, triggered by interactions between their ECD and specific ligands [29,72,79]. Given that many aGPCRs remain orphan receptors with no identified ligands, it is intriguing to speculate that additional aGPCRs, beyond GPR97 and GPR124, could play a role in aGPCR-GPCR transactivation. Moreover, the critical “trans-activating” role of aGPCR-ECD raises questions about the necessity of GPS-dependent proteolysis and aGPCR-mediated intracellular signaling in these processes. These observations are particularly intriguing because not all aGPCRs undergo GPS proteolysis, and some aGPCRs have alternatively spliced isoforms that consist solely of the N-terminal ECD, entirely lacking the 7TM domain [29,75,113]. It remains to be explored whether GPS cleavage-deficient aGPCRs or those with N-terminal ECD-only isoforms also play a role in the transactivation of other GPCRs.

While this review primarily focuses on the role of aGPCRs in the transactivation of different GPCRs, their involvement in traditional GPCR transactivation processes, such as the activation of RTKs, RSTKs, and other receptor types, should not be overlooked. Recent advances have revealed that some aGPCRs play an active role in mediating the activation of various receptor families, including the direct interaction and functional modulation of the teneurin and FLRT receptors by latrophilins, as well as TGFBR1 activation by GPR56 [114,115,116]. In conclusion, aGPCRs are versatile molecules that operate through various mechanisms, including the transactivation of different GPCR and receptor families.

## Data Availability

Not applicable.
